# The C allele of the rs741301 polymorphism in the *ELMO1* gene is associated with increased risk of diabetic retinopathy in patients with type 2 diabetes mellitus

**DOI:** 10.20945/2359-4292-2024-0283

**Published:** 2025-03-24

**Authors:** Luciane Moretto, Letícia de Almeida Brondani, Eliandra Girardi, Anna Carolina Meireles Vieira, Natália Emerim Lemos, Marilu Fiegenbaum, Luís Henrique Canani, Daisy Crispim, Cristine Dieter

**Affiliations:** 1 Serviço de Endocrinologia do Hospital de Clínicas de Porto Alegre, Porto Alegre, RS, Brasil; 2 Programa de Pós-graduação em Ciências Médicas: Endocrinologia, Faculdade de Medicina, Departamento de Clínica Médica, Universidade Federal do Rio Grande do Sul, Porto Alegre, RS, Brasil; 3 Unidade de Pesquisa Laboratorial, Centro de Pesquisa Experimental, Hospital de Clínicas de Porto Alegre, Porto Alegre, RS, Brasil; 4 Departamento de Bioquímica, Instituto de Química, Universidade de São Paulo, São Paulo, SP, Brasil; 5 Programa de Pós-graduação em Biociências, Universidade Federal de Ciências da Saúde de Porto Alegre, Porto Alegre, RS, Brasil

**Keywords:** Diabetic retinopathy, polymorphisms, *ELMO1*

## Abstract

**Objective:**

To investigate the association of the rs741301 polymorphism in the *ELMO1* gene with diabetic retinopathy (DR) in patients with type 2 diabetes mellitus (T2DM).

**Materials and methods:**

This study analyzed 350 patients withT2DM and DR (cases) and 234 patients withT2DM without this complication but with more than 10 years of diabetes mellitus (DM) (controls). DR was diagnosed by indirect fundoscopy. Genotyping was performed by allelic discrimination real-time PCR.

**Results:**

The frequency of the C/C genotype of the rs741301 polymorphism in the *ELMO1* gene was 26.9% in cases and 17.9% in controls (P = 0.011). After adjustment for covariables, the C/C genotype was associated with an increased risk of DR [odds ratio (OR) = 1.805, 95%CI 1.101–2.961; P = 0.019]. This association remained significant in dominant and additive inheritance models after adjustment for the same variables [OR = 1.597, 95%CI 1.089-2.343; P = 0.017; and OR = 1.818, 95%CI 1.099-3.007; P = 0.020].

**Conclusion:**

This study demonstrated an association between the presence of the C allele of the *ELMO1* rs741301 polymorphism and an increased risk of DR in patients with T2DM from Southern Brazil.

## INTRODUCTION

Diabetic retinopathy (DR) is a major microvascular complication of diabetes mellitus (DM), affecting approximately one-third of patients with DM (1. Despite being insidious and asymptomatic in its early stages, DR can advance over the years, potentially leading to irreversible vision impairment (^1,2^). The development and progression of DR are influenced by a complex interplay of clinical, environmental, and genetic factors (2.

Among the genetic factors, several single nucleotide polymorphisms (SNPs) in different genes have been identified as associated with DR [reviewed in (^3^)]. In this context, SNPs in the *TGFB1* (^4^), *TIE2* (^5^), *ANGPT1* (^5^), and *BDKRB1* (^6^) genes were previously reported to be associated with protection against DR, while SNPs in *ANGPT2* (^7^), *VEGFA* (^8^), *IL10* (^9^), *TNF* (^10^), *UCP1* (^11^), and *UPC2* (^12^) genes were associated with an increased risk of DR. However, these findings provide only partial insight into the DR development. Hence, identifying new genetic factors linked to this complication could advance our understanding of its progression.

The *engulfment and cell motility 1* (*ELMO1*) gene encodes a member of engulfment and cell motility protein family, which plays a role in various processes such as apoptotic cell phagocytosis (^13^), fibroblast migration (^14^), cytoskeleton reorganization (^15^), and lymphocyte infiltration (^16^) by interacting with the Dock180 protein. The rs741301 SNP in the *ELMO1* gene has been associated with increased risk of diabetic kidney disease (DKD) in different populations (^17,18,19,20,21,22,23^). ELMO1 appears to contribute to renal damage by being involved in angiogenesis and in the accumulation of oxidative stress (^24,25^). Since angiogenesis and increased oxidative stress are common pathways in DKD and DR (^26^), *ELMO1* could also be considered a potential candidate gene for DR. Therefore, this study aimed to investigate the association between the rs741301 SNP in the *ELMO1* and DR in patients with type 2 DM (T2DM) from a Southern Brazilian population, given the lack of studies examining the role of *ELMO1* in the DR pathogenesis.

## MATERIALS AND METHODS

### DR patients, phenotype measurements, and laboratory analyses

This case-control study was designed following STROBE and STREGA guidelines for reporting genetic association studies (^27,28^). The study population consisted of 584 type 2 DM (T2DM) patients, divided into 350 cases with DR and 234 controls without this complication. Only patients diagnosed with DM for at least 10 years were included in the control group. All participants were recruited from the outpatient clinic at the *Hospital de Clínicas de Porto Alegre* (Rio Grande do Sul, Brazil) from January 2005 to December 2013 (^12,29^). The research protocol was approved by the Ethics Committee in Research at *Hospital de Clínicas de Porto Alegre*, and all subjects provided written informed consent prior to the inclusion in the study.

Patients were diagnosed with T2DM according to the American Diabetes Association guidelines (30. DR was assessed by an experienced ophthalmologist using indirect fundoscopy through dilated pupils. DR was classified as “absent DR” (no fundus abnormalities), “non-proliferative DR” (NPDR; presence of microaneurysms, intraretinal hemorrhages, and hard exudates), or “proliferative DR” (PDR; newly formed blood vessels and/or growth of fibrous tissue into the vitreous cavity). DR classification was based on the most severely affected eye, according to the Global Diabetic Retinopathy Group scale (31. Patients without available DNA samples or with insufficient information for DR classification were excluded from this study.

A standard questionnaire was used to collect information on age, age at DM diagnosis, type and duration of DM, and drug treatment. Additionally, all patients underwent comprehensive physical and laboratory evaluations, as previously reported by our group (^12,29^). Ethnicity was defined based on self- classification, and only white subjects were included in the study. Serum and plasma samples were collected for laboratory analyses after 12h of fasting. Glucose levels were determined using the glucose oxidase method. Glycated hemoglobin (HbA1c) levels were measured by various methods, with results traceable to the Diabetes Control and Complications Trial (DCCT) method with offline calibration or using a conversion formulae (^32^).

### Genotyping

Total DNA was extracted from peripheral blood samples using a standardized technique. The *ELMO1* rs741301 SNP were genotyped using TaqMan SNP Genotyping Assays 20X (Thermo Fisher Scientific, Foster City, CA, USA; Assay ID: C_2672066_1_). Real-Time PCR reactions were performed in 384-well plates with a total volume of 5 µL, containing 2 ng of DNA, TaqMan Genotyping Master Mix 1X (Thermo Fisher Scientific), and TaqMan Genotyping Assay 1X. PCR reactions were conducted in a ViiA7 Real-Time PCR System (Thermo Fisher Scientific).

### Statistical analyses

Genotype and allele frequencies of the *ELMO1* rs741301 SNP were estimated by direct allele count- ing, and the Hardy-Weinberg Equilibrium (HWE) was tested using the chi-square (χ2) test. Allele and geno- type frequencies were compared between groups of subjects using χ2 tests. Moreover, genotypes were com- pared between groups of patients considering different inheritance models, categorized as previously suggested in: recessive (0: TT-TC *vs.* 1: CC), dominant (0: TT *vs.* 1: TC-CC), and additive (0: TT *vs.* 1: C/C) (^33^).

Normal distributions of quantitative clinical and laboratory variables were assessed using Kolmogorov- Smirnov and Shapiro-Wilk tests. Variables with normal distribution are presented as mean ± SD, while those with skewed distribution were log-transformed before analysis and are shown as median (25th-75th percentile values). Categorical data are presented as percentage. Clinical and laboratory characteristics were compared between case and control groups using Student’s t-test or χ2 tests.

The magnitude of association between the *ELMO1* rs741301 SNP and DR was estimated using odds ratios (OR) with 95% confidence intervals (CI). Multivariate logistic regression analyses were conducted to evaluate the independent association of the SNP of interest with DR, adjusting for possible confounding factors. Statistical analyses were performed using SPSS 18.0 software (SPSS, Chicago, IL), and P values < 0.05 were considered significant.

## RESULTS

### Sample description

The main characteristics of T2DM patients with and without DR are shown in Table 1. The mean age was higher in the control group compared to the case group (69.8 ± 10.5 *vs*. 66.2 ± 10.2; P < 0.0001). The proportion of males was higher in the case group than in the control group (55.1% *vs*. 40.3%; P = 0.001). As expected, the prevalence of DKD was higher in cases with DR compared to controls (78.0% *vs*. 53.6%; P < 0.0001).

**Table 1:** Clinical and laboratory characteristics of T2DM patients categorized according to the presence of DR

Characteristics	Controls (n = 234)	Cases with DR (n = 350)	P*
Age (years)	69.8 ± 10.5	66.2 ± 10.2	<0.0001
DM duration (years)	49.3 ± 10.5	45.3 ± 10.6	<0.0001
Gender (% males)	40.3	55.1	0.001
HbA1c (%)	7.3 ± 1.9	7.5 ± 1.8	0.111
BMI (kg/m2)	28.6 ± 5.2	28.2 ± 5.1	0.310
Arterial hypertension (%)	84.5	90.3	0.061
Triglycerides (mg/dL)	142.0 (106.0-213.0)	145.0 (101.0-208.0)	0.881
Total cholesterol (mg/dL)	191.9 ± 53.9	193.6 ± 51.3	0.715
LDL cholesterol (mg/dL)	109.2 ± 46.5	115.6 ± 45.3	0.140
HDL cholesterol (mg/dL)	46.5 ± 12.4	13.7 ± 12.0	0.008
DKD (%)	53.6	78.0	<0.0001

Variables are shown as mean ± SD, median (25th-75th percentiles) or %. * P values were computed using Student’s t or χ2 tests, as appropriate. BMI: body mass index; DR: diabetic retinopathy; HbA1c: glycated hemoglobin; T2DM: type 2 diabetes mellitus.

### Genotype and allele frequencies

Table 2 describes allele and genotype frequencies of the rs741301 SNP in the *ELMO1* gene in both case and control groups. Genotype distributions of the rs741301 SNP were consistent with the HWE in the control group (P ≥ 0.05). The frequency of the C/C genotype was higher in T2DM patients with DR compared to T2DM controls (26.9% *vs*. 17.9%, P = 0.011). Similarly, the frequency of the C allele was higher in cases than in controls (48% *vs*. 39%; P = 0.001). Additionally, this difference was significant under the recessive (P = 0.017), additive (P = 0.004), and dominant (P = 0.016) genetic models.

**Table 2:** Genotype and allele frequencies of the ELMO1 rs741301 SNP in T2DM patients categorized according to the presence of DR

ELMO1 rs741301 SNP	Controls (n = 234)	Cases (n = 350)	Unadjusted P*	Adjusted OR (95% IC)/P†
Genotype				
T/T	95 (40.6)	107 (30.5)	0.011	1
T/C	97 (41.5)	149 (42.6)		1.496 (0.987-2.269)/0.058
C/C	42 (17.9)	94 (26.9)		1.805 (1.101-2.961)/0.019
Allele				
T	0.61	0.52	0.001	
C	0.39	0.48		
Recessive model				
T/T + T/C	192 (82.1)	256 (73.1)	0.017	1
C/C	42 (17.9)	94 (26.9)		1.443 (0.931-2.237)/0.101
Additive model				
T/T	95 (69.3)	107 (53.2)	0.004	1
C/C	42 (30.7)	94 (46.8)		1.818 (1.099-3.007)/0.020
Dominant model				
T/T	95 (40.6)	107 (30.6)	0.016	1
T/C + C/C	139 (59.4)	243 (69.4)		1.597 (1.089-2.343)/0.017

Data are shown as number (%) or proportion. * P values were calculated using χ2 tests. † P values and ORs (95% CI) were obtained using logistic regression analyses adjusting for age, age at DM diagnosis, gender, HDL cholesterol, and presence of DKD.

After adjustment for age, gender, age at DM diagnosis, HDL cholesterol levels, and the presence of DKD, the C/C genotype of the rs741301 SNP was associated with increased risk of DR (OR = 1.805, 95%CI 1.101–2.961; P = 0.019). This association was also observed under the additive and dominant genetic models adjusting for the same covariates (additive model: OR = 1.818, 95%CI 1.099-3.007; P = 0.020; dominant model: OR = 1.597, 95%CI 1.089-2.343; P = 0.017).

Interestingly, when we stratified patients according to the DR severity, the frequency of the C/C genotype was 17.9% in the control group, 25.3% in the NPDR group, and 29.1% in the PDR group (P = 0.042). No difference was found when comparing the frequency of the C/C genotype between patients with NPDR and patients with PDR (25.3% *vs*. 29.1%, respectively; P = 0.711).

## DISCUSSION

This study aimed to investigate the frequency of the *ELMO1* rs741301 SNP in patients with T2DM stratified by the presence of DR. We demonstrated, for the first time, an association between the rs741301 C allele and an increased risk of DR in patients with T2DM from Southern Brazil. The association between the *ELMO1* rs741301 SNP and the risk of DR in patients with T2DM is plausible, with a probable relation to DR by the gene’s involvement in angiogenesis and oxidative stress pathways (^26^).

Supporting this, an experimental study using zebrafish demonstrated a decrease in vascular formation in the retina of *Elmo1*-/- zebrafish (^25^). Moreover, an observed increase in vessel thickness in larval hyaloids further suggests an impact of *Elmo1* on vessel structure, broadening its regulatory capacity in the vasculature (^25^). Therefore, we hypothesized that the rs741301 SNP in the *ELMO1* gene may alter *ELMO1* expression and consequently affect its function in vascular development.

Although no study to date has reported the involvement of *ELMO1* in the development of DR or analyzed the SNP in patients with DR, several studies have reported an association between the rs741301 SNP in *ELMO1* gene and an increased risk of DKD. In this context, it is important to highlight that DKD and DR share several common pathways, including the accumulation of oxidative stress due to chronic hyperglycemia, attributed to five main mechanisms: increased formation of advanced glycation end- products; elevated expression of the receptor for advanced glycation end-products; activation of protein kinase C isoforms; increased glucose flux in the polyol pathway; and upregulation of the hexosamine pathway (^34^). Additionally, Fang and cols. (^35^) conducted a two-sample mendelian randomization study from the perspective of genetics to assess the causal relation between DR and DKD. Data from twenty polymorphisms associated with DR from the FinnGen Consortium were tested regarding their associations with DKD. The authors showed positive associations of 20 genetically predicted DR polymorphisms with risk of DKD. Thus, polymorphisms associated with DKD may also be associated with DR.

Regarding the association of the rs741301 SNP in *ELMO1* and DKD, Bayoumy and cols. (^17^) found that the C/C genotype of this SNP was associated with a higher risk of DKD (OR = 2.7, 95%CI 1.4- 5.3; P = 0.016) in a study involving 400 Egyptian patients with DM, half of whom had DKD. Similarly, in Chinese patients with T2DM, the presence of the C allele of the rs741301 SNP was associated with an increased risk of DKD (OR = 1.75, 95%CI 1.19-2.28; P < 0.001) (^20^). This finding was consistent with a study conducted in the Iranian population, in which the presence of the C allele was associated with an elevated risk of DKD (OR = 2.5, 95%CI 1.2-5.4; P = 0.010) (^22^). In contrast, Bodhini and cols. (^18^) reported the association of the T/T genotype with an increased risk of DKD (OR = 1.48, 95%CI 1.02-2.55; P = 0.035) in patients with T2DM from a South Indian population. However, no association between the rs741301 SNP and DKD was found in studies conducted in Polish (^21^), Chinese (^23^), and Egyptian (^19^) populations.

Despite inconclusive results, *ELMO1* appears to play a role in DKD pathogenesis. Hathaway and cols. (^24^) demonstrated that the severity of renal fibrosis and urinary albumin excretion levels correlated with *Elmo1* expression values in Akita mice with genetically induced different levels of this gene. Additionally, higher expression of *Elmo1* correspondingly led to an increase in the levels of reactive oxygen species, indicating the potential involvement of this gene in renal damage by increased oxidative stress (^24^). Moreover, *ELMO1* was also described as a promotor of angiogenesis and early vascular development in zebrafish (^25^).

While our results contribute to a better understanding of the involvement of genetic polymorphisms in the pathogenesis of DR, a few limitations should be considered when interpreting our findings. First, there is the possibility of population stratification bias in our sample despite our focus on white participants and recruitment from the same hospitals for both cases and controls, which aimed to minimize the risk of false-positive/negative associations due to this bias. Second, our study represents the first demonstration of an association between the *ELMO1* rs741301 SNP and the risk of DR, yet we did not conduct a replication of this observed association in another Brazilian sample. Third, there is a lack of information regarding how the rs741301 SNP affects *ELMO1* expression and its functional role on DR susceptibility. Therefore, additional genetic studies are warranted to confirm the association between the rs741301 SNP in the *ELMO1* and the risk of DR in different ethnicities and populations. Moreover, functional studies are essential for a deeper understanding of how the rs741301 SNP influences *ELMO1* expression and activity.

In conclusion, this study provides the first evidence of an association between the C allele of the rs741301 SNP in the *ELMO1* gene and the risk of DR. This association appears to be plausible given the known involvement of ELMO1 in vascular formation, angiogenesis, and the accumulation of reactive oxygen species. Further studies are needed to validate these findings in other populations.

Acknowledgments: this study was partially financially supported by *Conselho Nacional Desenvolvimento Científico e Tecnológico (CNPq), Financiamento e Incentivo à Pesquisa* (Fipe) at *Hospital de Clínicas de Porto Alegre* (number 2022-0383), *Fundação de Amparo à Pesquisa do Estado do Rio Grande do Sul* (Fapergs; number 1928-2551/13-2), and *Coordenação de Aperfeiçoamento de Pessoal de Nível Superior* (Capes). Cristine Dieter, Daisy Crispim, Luciane Moretto, and Luís Henrique Canani received scholarships from CNPq, while Eliandra Girardi received a scholarship from Fapergs.

Disclosure: no potential conflict of interest relevant to this article was reported.

## References

[1] Sun H, Saeedi P, Karuranga S, Pinkepank M, Ogurtsova K, Duncan BB (2022). IDF Diabetes Atlas: Global, regional and country-level diabetes prevalence
estimates for 2021 and projections for 2045.. Diabetes Res Clin Pract.

[2] Wong TY, Cheung CM, Larsen M, Sharma S, Simo R. (2016). Diabetic retinopathy.. Nat Rev Dis Primers.

[3] Bhatwadekar AD, Shughoury A, Belamkar A, Ciulla TA. (2021). Genetics of Diabetic Retinopathy, a Leading Cause of Irreversible Blindness
in the Industrialized World.. Genes (Basel).

[4] Costa AR, Dieter C, Canani LH, Assmann TS, Crispim D. (2023). The rs1800469 T/T and rs1800470 C/C genotypes of the TGFB1 gene confer
protection against diabetic retinopathy in a Southern Brazilian
population.. Genet Mol Biol.

[5] Dieter C, Lemos NE, de Faria Correa NR, Assmann TS, Pellenz FM, Canani LH (2023). Polymorphisms in TIE2 and ANGPT-1 genes are associated with protection
against diabetic retinopathy in a Brazilian population.. Arch Endocrinol Metab.

[6] Brondani LA, Crispim D, Pisco J, Guimaraes JA, Berger M. (2019). The G Allele of the rs12050217 Polymorphism in the BDKRB1 Gene Is
Associated with Protection for Diabetic Retinopathy.. Curr Eye Res.

[7] Dieter C, Lemos NE, de Faria Correa NR, Costa AR, Canani LH, Crispim D (2021). The rs2442598 polymorphism in the ANGPT-2 gene is associated with risk for
diabetic retinopathy in patients with type 1 diabetes mellitus in a Brazilian
population.. Arch Endocrinol Metab.

[8] Valiatti FB, Crispim D, Benfica C, Valiatti BB, Kramer CK, Canani LH. (2011). [The role of vascular endothelial growth factor in angiogenesis and
diabetic retinopathy].. Arq Bras Endocrinol Metabol.

[9] da Silva Pereira BL, Polina ER, Crispim D, Sbruzzi RC, Canani LH, Dos Santos KG. (2018). Interleukin-10 -1082A > G (rs1800896) polymorphism is associated with
diabetic retinopathy in type 2 diabetes.. Diabetes Res Clin Pract.

[10] Sesti LF, Crispim D, Canani LH, Polina ER, Rheinheimer J, Carvalho PS (2015). The -308G>a polymorphism of the TNF gene is associated with
proliferative diabetic retinopathy in Caucasian Brazilians with type 2
diabetes.. Invest Ophthalmol Vis Sci.

[11] Brondani LA, de Souza BM, Duarte GC, Kliemann LM, Esteves JF, Marcon AS (2012). The UCP1 -3826A/G polymorphism is associated with diabetic retinopathy and
increased UCP1 and MnSOD2 gene expression in human retina.. Invest Ophthalmol Vis Sci.

[12] Crispim D, Fagundes NJ, dos Santos KG, Rheinheimer J, Boucas AP, de Souza BM (2010). Polymorphisms of the UCP2 gene are associated with proliferative diabetic
retinopathy in patients with diabetes mellitus.. Clin Endocrinol (Oxf).

[13] deBakker CD, Haney LB, Kinchen JM, Grimsley C, Lu M, Klingele D (2004). Phagocytosis of apoptotic cells is regulated by a UNC-73/ TRIO-MIG-2/RhoG
signaling module and armadillo repeats of CED-12/ELMO.. Curr Biol.

[14] Grimsley CM, Kinchen JM, Tosello-Trampont AC, Brugnera E, Haney LB, Lu M (2004). Dock180 and ELMO1 proteins cooperate to promote evolutionarily conserved
Rac-dependent cell migration.. J Biol Chem.

[15] Sanui T, Inayoshi A, Noda M, Iwata E, Stein JV, Sasazuki T (2003). DOCK2 regulates Rac activation and cytoskeletal reorganization through
interaction with ELMO1.. Blood.

[16] Janardhan A, Swigut T, Hill B, Myers MP, Skowronski J. (02004). HIV-1 Nef binds the DOCK2-ELMO1 complex to activate rac and inhibit
lymphocyte chemotaxis.. PLoS Biol.

[17] Bayoumy NMK, El-Shabrawi MM, Leheta OF, Abo El-Ela AEM, Omar HH. (2020). Association of ELMO1 gene polymorphism and diabetic nephropathy among
Egyptian patients with type 2 diabetes mellitus.. Diabetes Metab Res Rev.

[18] Bodhini D, Chidambaram M, Liju S, Revathi B, Laasya D, Sathish N (2016). Association of rs11643718 SLC12A3 and rs741301 ELMO1 Variants with Diabetic
Nephropathy in South Indian Population.. Ann Hum Genet.

[19] El Nahid MS, Al-Ganiny AFM, Youssef RN. (2022). Association between engulfment and cell motility 1-gene polymorphisms and
diabetic nephropathy in an Egyptian population with type 2 diabetes.. J Diabetes Metab Disord.

[20] Hou Y, Gao Y, Zhang Y, Lin ST, Yu Y, Yang L. (2019). Interaction between ELMO1 gene polymorphisms and environment factors on
susceptibility to diabetic nephropathy in Chinese Han population.. Diabetol Metab Syndr.

[21] Kwiendacz H, Nabrdalik K, Adamczyk P, Moczulski D, Moczulska H, Trautsolt W (2020). Association of single nucleotide polymorphism (rs741301) of the ELMO1 gene
with diabetic kidney disease in Polish patients with type 2 diabetes: a pilot
study.. Endokrynol Pol.

[22] Mehrabzadeh M, Pasalar P, Karimi M, Abdollahi M, Daneshpour M, Asadolahpour E (2016). Association between ELMO1 gene polymorphisms and diabetic nephropathy in an
Iranian population.. J Diabetes Metab Disord.

[23] Wu HY, Wang Y, Chen M, Zhang X, Wang D, Pan Y (2013). Association of ELMO1 gene polymorphisms with diabetic nephropathy in
Chinese population.. J Endocrinol Invest.

[24] Hathaway CK, Chang AS, Grant R, Kim HS, Madden VJ, Bagnell CR Jr (2016). High Elmo1 expression aggravates and low Elmo1 expression prevents diabetic
nephropathy.. Proc Natl Acad Sci U S A.

[25] Boger M, Bennewitz K, Wohlfart DP, Hausser I, Sticht C, Poschet G (2022). Comparative Morphological, Metabolic and Transcriptome Analyses in elmo1
(-/-) , elmo2 (-/-) , and elmo3 (-/-) Zebrafish Mutants Identified a Functional
Non-Redundancy of the Elmo Proteins.. Front Cell Dev Biol.

[26] Thomas MC, Brownlee M, Susztak K, Sharma K, Jandeleit-Dahm KA, Zoungas S (2015). Diabetic kidney disease.. Nat Rev Dis Primers.

[27] Little J, Higgins JP, Ioannidis JP, Moher D, Gagnon F, von Elm E (2009). STrengthening the REporting of Genetic Association Studies (STREGA)--an
extension of the STROBE statement.. Genet Epidemiol.

[28] von Elm E, Altman DG, Egger M, Pocock SJ, Gotzsche PC, Vandenbroucke JP (2008). The Strengthening the Reporting of Observational Studies in Epidemiology
(STROBE) statement: guidelines for reporting observational studies.. J Clin Epidemiol.

[29] Dieter C, Lemos NE, Girardi E, Ramos DT, Pellenz FM, Canani LH (2023). The rs3931283/PVT1 and rs7158663/MEG3 polymorphisms are associated with
diabetic kidney disease and markers of renal function in patients with type 2 diabetes
mellitus.. Mol Biol Rep.

[30] ElSayed NA, Aleppo G, Aroda VR, Bannuru RR, Brown FM, Bruemmer D (2023). 2. Classification and Diagnosis of Diabetes: Standards of Care in
Diabetes-2023. Diabetes Care.

[31] Wilkinson CP, Ferris FL 3rd, Klein RE, Lee PP, Agardh CD, Davis M (2003). Proposed international clinical diabetic retinopathy and diabetic macular
edema disease severity scales.. Ophthalmology.

[32] Camargo JL, Zelmanovitz T, Paggi A, Friedman R, Gross JL. (1998). Accuracy of conversion formulae for estimation of
glycohaemoglobin. Scand J Clin Lab Invest.

[33] Zintzaras E, Lau J. (2008). Synthesis of genetic association studies for pertinent gene-disease
associations requires appropriate methodological and statistical
approaches.. J Clin Epidemiol.

[34] Giacco F, Brownlee M. (2010). Oxidative stress and diabetic complications.. Circ Res.

[35] Fang J, Luo C, Zhang D, He Q, Liu L. (2023). Correlation between diabetic retinopathy and diabetic nephropathy: a
two-sample Mendelian randomization study.. Front Endocrinol (Lausanne).

